# Replication-deficient whole-virus vaccines against cytomegalovirus induce protective immunity in a guinea pig congenital infection model

**DOI:** 10.1128/jvi.00207-25

**Published:** 2025-06-11

**Authors:** Mark R. Schleiss, Claudia Fernández-Alarcón, Craig J. Bierle, Adam P. Geballe, Alexey Badillo-Guzman, Christine E. Tanna, Kanokpan Tsriwong, Mark Blackstad, Jian Ben Wang, Michael A. McVoy

**Affiliations:** 1 Department of Pediatrics, University of Minnesota Medical School 12269, Minneapolis, Minnesota, USA; 2 Fred Hutchinson Cancer Center 7286, Seattle, Washington, USA; 3 Department of Pediatrics, Virginia Commonwealth University School of Medicine 466504, Richmond, Virginia, USA; Lerner Research Institute, Cleveland Clinic, Cleveland, Ohio, USA

**Keywords:** cytomegalovirus, congenital cytomegalovirus, cytomegalovirus vaccine, DISC vaccine, Guinea pig

## Abstract

Vaccines are needed to prevent congenital human cytomegalovirus (HCMV) infections. This study used the guinea pig cytomegalovirus (GPCMV) model to examine replication-deficient whole-virus vaccines for protection against maternal viremia and congenital CMV infection. Two recombinant GPCMVs, GP51-DD and GP52-DD, were engineered with destabilization domains fused to the essential viral late proteins GP51 and GP52. These viruses, predicted to replicate in the presence of the synthetic ligand Shield-1 but not in its absence, were evaluated for Shield-1 dependence *in vitro* and for safety, immunogenicity, and efficacy in the GPCMV model. GP52-DD was profoundly Shield-1-dependent, producing no detectable infectious progeny in its absence. In contrast, the replication of GP51-DD was delayed in the absence of Shield-1 but reached similar peak titers with or without the compound. GPCMV-seronegative guinea pigs received two subcutaneous injections of phosphate-buffered saline, GP51-DD, GP52-DD, or wild-type GPCMV (WT-GPCMV). DNAemia attributable to vaccination was noted in 10/10 (100%) of WT-GPCMV-immunized animals but in only 10/28 animals (36%) immunized with DD vaccines (*P* < 0.001). GPCMV-specific ELISA and interferon-gamma ELISpot responses were similar in all vaccinated groups. When immunized animals were bred and challenged during pregnancy with virulent GPCMV, DNAemia was detected in all sham-immunized controls and in 44% of GP52-DD-immunized dams (at significantly reduced levels) but was absent in dams immunized with GP51-DD or WT-GPCMV. Immunization with GP52-DD, GP51-DD, or WT-GPCMV significantly reduced congenital GPCMV transmission compared to placebo (protective efficacies of 89, 94, and 100%, respectively). Thus, replication-impaired GP51-DD and replication-deficient GP52-DD vaccines were comparable to WT-GPCMV in immunogenicity and protective efficacy.

**IMPORTANCE:**

Congenital HCMV infections could potentially be prevented by a vaccine, but most vaccines that have advanced in clinical trials have been modestly effective, at best. Subunit HCMV vaccines chiefly target envelope glycoproteins, but none has proven effective at engendering durable protective immunity. A vaccine that confers immune responses to a broader repertoire of immunogens than a subunit vaccine, such as a whole-virus, live-attenuated vaccine, could confer improved protection. However, there are safety concerns for live-attenuated HCMV vaccines. Using the GPCMV model of congenital infection, this study demonstrates that two replication-impaired whole virus vaccines, though attenuated in animals, are highly immunogenic and induce preconception immunity that protects against maternal viremia and fetal infection after wild-type GPCMV challenge during pregnancy.

## INTRODUCTION

Human cytomegalovirus (HCMV) is a major cause of disability in newborns, and the development of an effective vaccine is a major public health priority (1–3). Because CMVs are highly species-specific, preclinical vaccine development must utilize animal viruses that are closely related to HCMV. While murine and rhesus macaque CMVs have been used for this purpose, guinea pig cytomegalovirus (GPCMV) is a particularly useful small animal model for vaccine development since the virus, unlike murine CMV, will infect the placenta and fetus after primary maternal infection during pregnancy (4–7). Thus, the guinea pig/GPCMV model has been utilized to examine a variety of vaccine designs and antiviral therapy strategies toward the goal of developing interventions that can prevent congenital HCMV infection (8–15).

HCMV vaccine development has largely focused on subunit vaccines based on glycoprotein B (gB), which mediates membrane fusion and entry into all cell types, and the pentameric complex (PC), a target for potent antibodies that selectively neutralize HCMV entry into endothelial and epithelial cells (3, 16–18). A recent vaccine candidate, V160, utilized a replication-defective recombinant virus in which essential viral proteins were fused to the FK506-binding protein 12-destabilization domain (DD) (19–21). V160 requires the presence of the synthetic ligand Shield-1, which does not exist in nature, to stabilize viral-encoded DD-fusion proteins and replicate in cell culture (21, 22). In V160, HCMV immediate early 1 (IE1), immediate early 2 (IE2), and pUL51 proteins are fused to DDs (21). However, the degradation of IE1 and IE2 proteins, which helps eliminate viral replication, likely also limits the production of viral antigens with early or late expression kinetics *in vivo* and thus may impair the overall immunogenicity of the vaccine.

To optimize a DD-based, replication-defective vaccine strategy in the GPCMV model of congenital infection, we generated two Shield-1-dependent recombinant GPCMVs, designated as GP51-DD and GP52-DD, where the essential late viral proteins GP51 or GP52 were fused to DDs. We evaluated the Shield-1 dependence of these viruses *in vitro* and tested their safety, immunogenicity, and efficacy against congenital GPCMV infection in a vaccination/pregnancy/challenge model. We found that the GP51-DD and GP52-DD viruses were differentially dependent on Shield-1 for replication in tissue culture. When used as replication-deficient vaccines, both viruses were highly attenuated and immunogenic in guinea pigs and protected against maternal and congenital GPCMV infection at levels comparable to prior infection by replication-competent wild-type GPCMV.

## MATERIALS AND METHODS

### Virus and cells

Viruses were propagated in guinea pig lung fibroblasts (GPL) (ATCC CCL-158, also known as JH4) maintained in F-12 medium and supplemented with 10% fetal calf serum (Fisher Scientific), 10,000 IU/L penicillin, 10 mg/L streptomycin (Gibco-BRL), and 0.075% NaHCO_3_ (Gibco-BRL). Growth curves, viral titers, and neutralization assays were performed as described previously (23). GPCMV stocks were prepared and titered by plaque assay using GPL cells, as previously described (24). Wild-type GPCMV (WT-GPCMV) consisted of a salivary gland-derived stock (serially passaged 24 times in Strain Two guinea pigs) corresponding to GPCMV strain 22122 and prepared as previously described (25). Shield-1 was the gift of Dai Wang (Merck Vaccines) and was used at a final concentration of 2 µM when cells were cultured for viral stock preparations. Titrations of GP51-DD and GP52-DD viruses were performed in the presence of 2 µM Shield-1.

### Construction of GP51-DD and GP52-DD viruses

The recombinant viruses GP51-DD and GP52-DD were derived by modifying the infectious bacterial artificial chromosome (BAC) clone N13R10r129-TurboFP635 (26). This clone was modified from BAC N13R10, a complete genomic clone of tissue culture-adapted GPCMV strain 22122 (23). N13R10 was first modified to restore an intact *gp129* open reading frame (ORF), which encodes a subunit of the GPCMV PC, generating BAC N13R10r129 (27), then further modified by insertion of a marker cassette encoding the red fluorescent protein (RFP) TurboFP635 to produce N13R10r129-TurboFP635 (26).

Two-step galactokinase-mediated recombineering was used, as described previously (27, 28) to construct viruses GP51-DD and GP52-DD. In step 1, a *galK* cassette encoding galactokinase was inserted into BAC N13R10r129-TurboFP635 between the first and second codons of ORFs *GP51* or *GP52* by PCR amplification of plasmid pgalK using the oligonucleotide pairs listed under step 1 in Table 1. These PCR products were transformed into *E. coli* strain SW102 cells containing BAC N13R10r129-TurboFP635, followed by colony selection on Gal-positive plates and verification of clones containing the expected *galK* insertions by PCR and targeted sequencing, as described previously (29). In step 2, oligonucleotide pairs (Table 1) were used to PCR-amplify DD-encoding sequences from plasmid pENTR4-FKBP12DD N-term A (AddGene, plasmid #17414), and the products were then transformed into SW102 cells containing the appropriate N13R10r129-TurboFP635 BACs with *galK* insertions in *GP51* or *GP52* that were constructed in step 1 (30). Colonies were then isolated using Gal counterselection plates, and clones containing the desired DD-encoding insertions were verified by PCR screening and confirmed by targeted Sanger sequencing (28).

**TABLE 1:** Oligonucleotides used for construction of GP51-DD and GP52-DD

Virus	Step	Oligonucleotide	Sequence
GP51-DD	1	GP51-N-galk-FW	GATACGGTACGCGACGGTACGGCGTGTCTCCTGCGTGCCGCGGGGCGATGACGACTCACTATAGGGCGAATTGG
		GP51-N-galk-RV	TCCTCCACATCCACGACATTCGCCTCGTCCGAAGAACCGTTCACGGCCGCGCTATGACCATGATTACGCCAAGC
	2	N-DD-GP51-FW	GATACGGTACGCGACGGTACGGCGTGTCTCCTGCGTGCCGCGGGGCGATGGGAGTGCAGGTGGAAACCATCT
		N-DD-GP51-RV	TCCTCCACATCCACGACATTCGCCTCGTCCGAAGAACCGTTCACGGCCGCTTCCGGTTTTAGAAGCTCCACA
	conf^*a*^	GP51-N-galk-FW-S	GATACGGTACGCGACGGTA
		GP51-N-galk-RV-out	ACAGTATGTCTTTGTGCGCG
GP52-DD	1	GP52-N-galk-FW	TCGCGACCCGTACGAGATCGGTTCCTAAAAACAGAATAAAGCCAACAATGACGACTCACTATAGGGCGAATTGG
		GP52-N-galk-RV	GGAGCGTCCATGTTTGAGAAATGATGACACCAGCCTTTGTATGTGAAATCGCTATGACCATGATTACGCCAAGC
	2	N-DD-GP52-FW	TCGCGACCCGTACGAGATCGGTTCCTAAAAACAGAATAAAGCCAACAATGGGAGTGCAGGTGGAAACCATCT
		N-DD-GP52-RV	GGAGCGTCCATGTTTGAGAAATGATGACACCAGCCTTTGTATGTGAAATCTTCCGGTTTTAGAAGCTCCACA
	conf^*a*^	GP52-N-galk-FW-out	TACCGTATCGCAAGATGAGC
		GP52-N-galk-RV-out	GGATCGTCTAAGGATACGTG

^*a*^ conf, primer pairs used for confirmation of DD sequence insertions.

**Fig 1:**
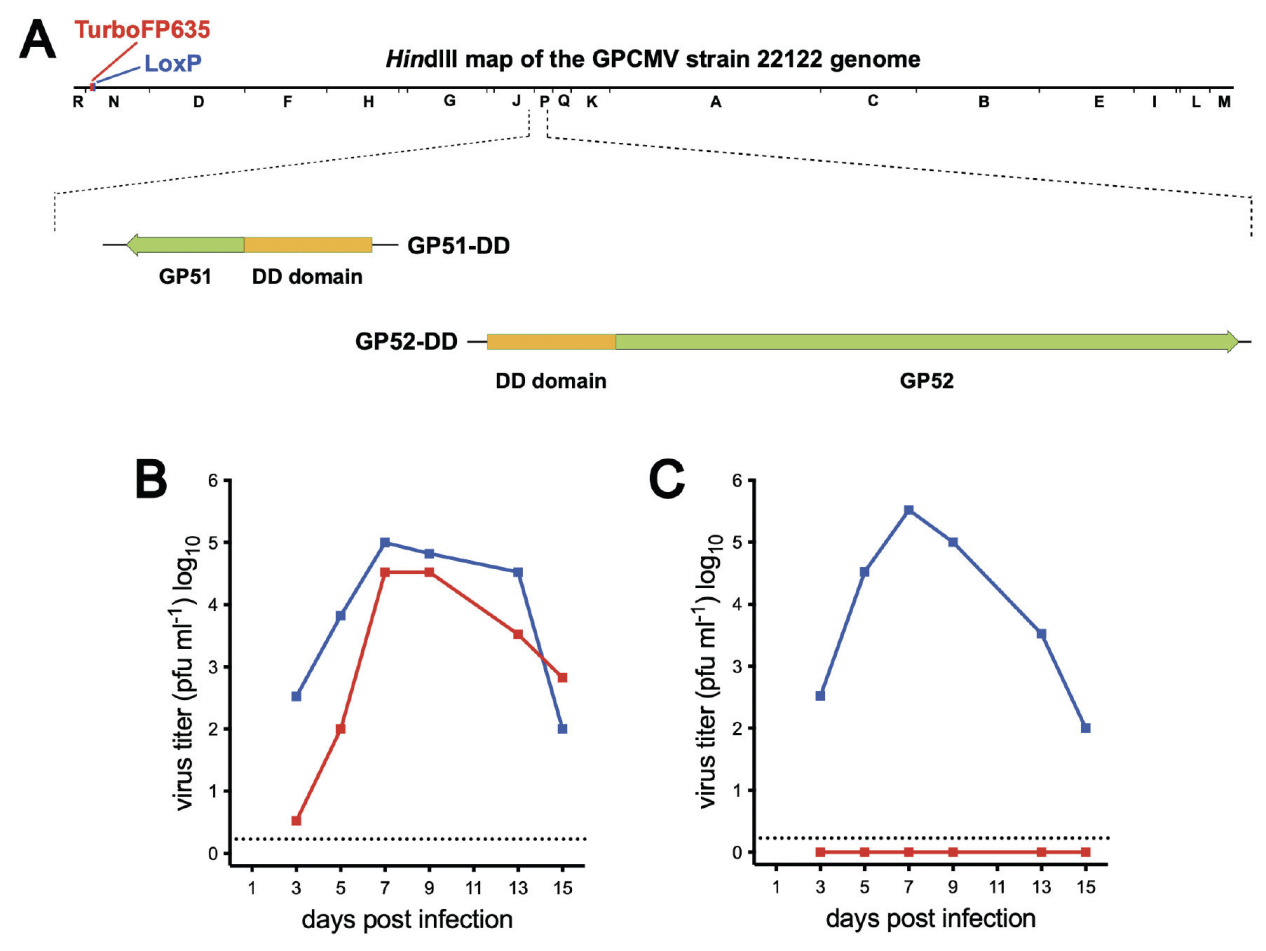
Design and *in vitro* growth properties of GP51-DD and GP52-DD recombinant GPCMVs. (**A**) *Hin*d III restriction map illustrating the locations of ORFs *GP51* and *GP52* in the GPCMV genome, as well as a marker gene cassette encoding the red fluorescent protein TurboFP635 and an adjacent *LoxP* site that is residual after cre excision of the BAC origin of replication. Expanded below are *GP51* and *GP52* ORFs that have been modified to encode N-terminal DD fusions to GP51 and GP52 in viruses GP51-DD and GP52-DD, respectively. Replicate GPL cultures were infected with (**B**) GP51-DD or (**C**) GP52-DD at an MOI of 0.01, then maintained for 15 days in medium either containing (blue) or lacking (red) 2 µM Shield-1. Samples of culture supernatants were collected on the days indicated, and infectious titers were determined in the presence of 2 µM Shield-1. The dashed line indicates the level of detection (LOD) of 1.7 PFU mL^−1^.

BAC-derived viruses GP51-DD and GP52-DD were reconstituted by transfection of BAC DNA into GPL cells using Effectene Transfection Reagent (Qiagen, Hilden, Germany), as previously described (31). Shield-1 (2 µM) was maintained in the culture medium during transfection, and during the subsequent generation of viral stocks. The correct genomic configuration of GP51-DD and GP52-DD viruses (Fig. 1A) was confirmed by PCR and sequence analyses of the DD domain sequences in the *GP51* or *GP52* ORFs using the primer pairs listed in Table 1.

### *In vitro* viral replication assays

Growth curve analyses were performed, as previously described (29). Replicate flasks of GPL cells were infected with GP51-DD or GP52-DD at a multiplicity of infection (MOI) of 0.01 and then maintained for 15 days in medium that either contained or lacked 2 µM Shield-1. Samples of culture supernatants were collected at 3, 5, 7, 9, and 13 days post-inoculation, and infectious titers were determined on fresh GPL cells in the presence of 2 µM Shield-1.

### Guinea pig immunizations

Outbred Hartley guinea pigs (200–300 g) were purchased from Elm Hill Laboratories (Chelmsford, MA). Guinea pigs were confirmed to be GPCMV-seronegative by enzyme-linked immunosorbent assay (ELISA), as previously described (32), prior to the initiation of the immunization studies. GPCMV-seronegative animals were randomly assigned to one of the four vaccine groups. Guinea pigs were immunized subcutaneously (SC) twice at 3-week intervals in the following group assignments: group 1 (*N* = 14), GP51-DD vaccine (dose, 5 × 10^6^ PFU); group 2 (*N* = 14), GP52-DD vaccine (dose, 5 × 10^6^ PFU); group 3 (*N* = 10), WT-GPCMV (dose, 1.4 × 10^5^ PFU); and group 4 (*N* = 10), sham-inoculated with phosphate-buffered saline (PBS). Serial weights and DNAemia were monitored in these animals to gauge for attenuation *in vivo* (described below). Two additional GPCMV-seronegative animals were sham-inoculated with PBS and utilized as controls for other subsequently performed immune assays (described below) but were not used for pregnancy/challenge studies.

### Immune assays

Blood samples were collected on days 7, 14, and 21 following each immunization; the day 21 bleed after the first dose of vaccine was obtained immediately before the second immunization was administered. Blood was also obtained from pregnant animals (described in “Real-time qPCR analysis,” below) immediately prior to the viral challenge, at days 7, 14, and 21 days post-challenge, and upon delivery.

GPCMV-specific IgG responses were reported as geometric mean titers (GMTs) determined as a reciprocal of endpoint dilutions in an ELISA using GPCMV viral particles as the coating antigen, as previously described (29). Samples negative at a 1:80 dilution were assigned a GMT value of 40 for statistical comparisons. GPCMV-reactive IgG avidity was measured, as previously described (10). To determine the avidity of GPCMV-specific IgG responses, the ELISA described above was conducted using serum dilutions of 1:160, 1:320, 1:640, and 1:1,280 in the presence or absence of 4 M urea. OD_450_ values for serum dilutions within the linear range of the ELISA (0.5–1.0 OD units) were used to calculate the IgG avidity index (AI) for each sample, defined as the average of the ratios for different dilutions of the OD_450_ values obtained with 4 M urea to those obtained without urea.

A recombinant GPCMV expressing RFP, N13R10r129-RFP, was used to assay for neutralizing antibody responses (24). Neutralizing titers were reported as reciprocal GMTs of the lowest serum dilutions that resulted in ≥50% reduction in the number of RFP-positive foci on GPL cells detected 72 h post-inoculation. Samples negative at a 1:40 dilution were assigned a GMT value of 20 for statistical comparisons.

To measure cell-mediated responses, IFN-γ ELISpot assays were performed. Mouse monoclonal anti-IFN-γ antibodies (N-G3 and V-E4) were a gift from Hubert Schäfer (33, 34). ELISpot assays were carried out as previously described, with minor modifications (35, 36). The following peptides corresponding to the immunodominant sequences of the T-cell target, GP83, were utilized: GP83 peptide 1, ACMTHVDSL; GP83 peptide 2, LASHAQVVM; GP83 peptide 3, LGIVHFFDN; and GP83 peptide 4, GDAKDDGSE. The peptides were purchased from Sigma Life Sciences (The Woodlands, Texas) and prepared according to the manufacturer’s specifications. A titration experiment demonstrated that a concentration of 1 µg peptide/well produced optimal ELISpot read-outs. Spleens were harvested from guinea pigs at 28 to 35 days following the second vaccination, and splenocytes were isolated over Ficoll gradients, as previously described (36). Isolated splenocytes (1 × 10^5^) were mixed with 50 µL stimulant (peptide at 1 µg/well) or controls (a no-stimulation, dimethyl sulfoxide negative control and a positive control using concanavalin A at 20 µg/mL). Secondary antibody and developing reagents were added, as previously described (36), and spots were counted with an AID ELISpot reader system using ELISpot 6.0-iSpot (Autoimmun Diagnostika GmbH, Straßberg, Germany). Multiple replicates were performed (*n* = 6 for PBS and WT-GPCMV groups and *n* = 12 for GP51-DD and GP52-DD groups) and analyzed for comparisons. To control for background in ELISpot analyses, the mean number of spots obtained in the presence of medium alone (no cells) was subtracted from the mean number of spots counted in each of the control or experimental conditions, as previously described (36).

### Breeding and challenge during pregnancy

To assay for the potential protective effect of vaccination against GPCMV transmission during pregnancy, most immunized female guinea pigs were bred with GPCMV-seronegative males for subsequent challenge studies; the animals that were not bred were retained for immunological studies, as described above (IFN-γ ELISpot), using harvested splenocytes that were obtained upon necropsy following completion of the vaccination series. These immunized animals were never pregnant. For the remaining dams, pregnancy was confirmed by measurement of progesterone ELISA, serial physical examination with palpation of developing fetuses, and prenatal ultrasonography (37). Pregnant dams were challenged at approximately 35 days gestation with 1 × 10^6^ PFU of a virulent salivary gland-adapted workpool of WT-GPCMV, which was administered by SC injection. Maternal blood samples were obtained at 7, 14, and 21 days post-challenge and again at delivery. Pregnancy outcomes were monitored, pup blood was obtained after delivery, and pup tissues (lung, liver, and spleen) were harvested and homogenized within 24 h of birth (following necropsy) for DNA extraction as described previously to assess for congenital infection (29). Viral DNA detection and viral load comparisons were carried out as described in the following section.

### Real-time qPCR analysis

Viral loads were quantified by a qPCR assay, as described previously (15). Briefly, DNA was extracted from either 100 µL of citrated blood or 0.05 g of homogenized tissues using the QIAamp 96 DNA QIAcube HT Kit (Qiagen). Amplification primers GP83TM_F1 (5′-CGTCCTCCTGTCGGTCAAAC-3′) and GP83TM_R1 (5′-CTCCGCCTTGAACACCTGAA-3′) were used at a final concentration of 0.4 µM, while the *GP83* hydrolysis probe (FAM-CGCCTGCATGACTCACGTCGA-BHQ1) was used at 0.1 µM. PCR was performed, and the data were analyzed, as previously described (15). DNAemia was expressed as the total number of genome copies per milliliter of blood. Viral loads in tissue were expressed as genome copies per milligram of tissue. PCR assays were considered positive if both replicates demonstrated amplifiable DNA or if one of two replicates was positive in two independent experiments. The limit of detection of the tissue assay was two genome copies/mg tissue and 200 copies/mL for the blood PCR, and negative values were assigned these values for statistical comparisons, as previously described (15). For the purpose of this study, congenital infection was defined as any positive PCR from pup blood or visceral organ (lung, liver, or spleen) found in samples obtained with 24 h of delivery.

### Statistical analyses

GraphPad Prism (version 10.0) was used for statistical analyses. Pup mortality, rates of DNAemia following immunization of non-pregnant animals, and transmission rate data were compared using Fisher’s exact test with one-sided comparisons. Log_10_-transformed antibody responses (ELISA titers, neutralization titers, and IgG avidity indices), as well as IFN−γ responses, were compared across groups and at individual time points by one-way analysis of variance (ANOVA) and *t*-test, as previously described (38). DNAemia, blood and visceral organ viral loads, and pup birth weight data were compared by ANOVA. Repeated-measures ANOVA was used to compare the means of animal groups when data points were measured and available for the same animals at multiple times across groups (serial weights, immune responses); for parameters where animals did not have consistently measurable results (i.e., no measurable viral load and with negative results for congenital infection by PCR), regular ANOVA was used.

## RESULTS

### Generation and *in vitro* characterization of Shield-1-dependent GPCMVs

The effectiveness with which different proteins are destabilized by DD fusion varies widely, as does the ability of Shield-1 to stabilize different DD fusions (22). Hence, creating a virus that replicates efficiently in the presence of Shield-1 and poorly or not at all in its absence may require the construction and characterization of several viruses encoding DD fusions to different essential viral proteins (39). The preclinical development of V160 involved evaluating at least 11 HCMV recombinants encoding DD fusions to different viral proteins, and among these transgenic viruses, an absolute requirement for Shield-1 for viral replication was only noted when the DD was fused to UL51 or UL52 (21). This observation led us to target GP51 and GP52, the GPCMV homologs of UL51 and UL52, for destabilization. GP51-DD and GP52-DD were designed such that N-terminal DDs were fused to each respective protein (Fig. 1A). PCR and Sanger sequencing were used to confirm the insertions of the DD sequences (data not shown). To assess for Shield-1-dependent replication of the GP51-DD and GP52-DD viruses *in vitro,* growth curves were performed in the presence or absence of Shield-1 (Fig. 1B and C). GP51-DD replicated in the absence or presence of Shield-1, reaching similar peak titers in both conditions, but its replication kinetics were delayed 2 to 3 days without Shield-1 (Fig. 1B). In contrast, the replication of GP52-DD was profoundly dependent on Shield-1, reaching peak titers of ~10^6^ PFU/mL in the presence of Shield-1, but with no infectious progeny detected in its absence (Fig. 1C).

### GP51-DD and GP52-DD are attenuated in guinea pigs

To evaluate the virulence and immunogenicity of GP51-DD and GP52-DD, outbred Hartley guinea pigs (>350 g, 25–31 days of age) were randomly assigned to one of the four groups. Guinea pigs were injected SC with GP51-DD or GP52-DD (*n* = 14; 5 × 10^6^ PFU/injection) or WT-GPCMV (*n* = 10; 1.4 × 10^5^ PFU/injection) or sham inoculated with PBS (*n* = 10). These inoculations were repeated after 21 days. Blood and serum were collected every 7 days post-inoculation so that viral loads and anti-GPCMV humoral responses could be quantified using qPCR, ELISA, and, at 21 days post-challenge, by determination of neutralization titers.

**Fig 2:**
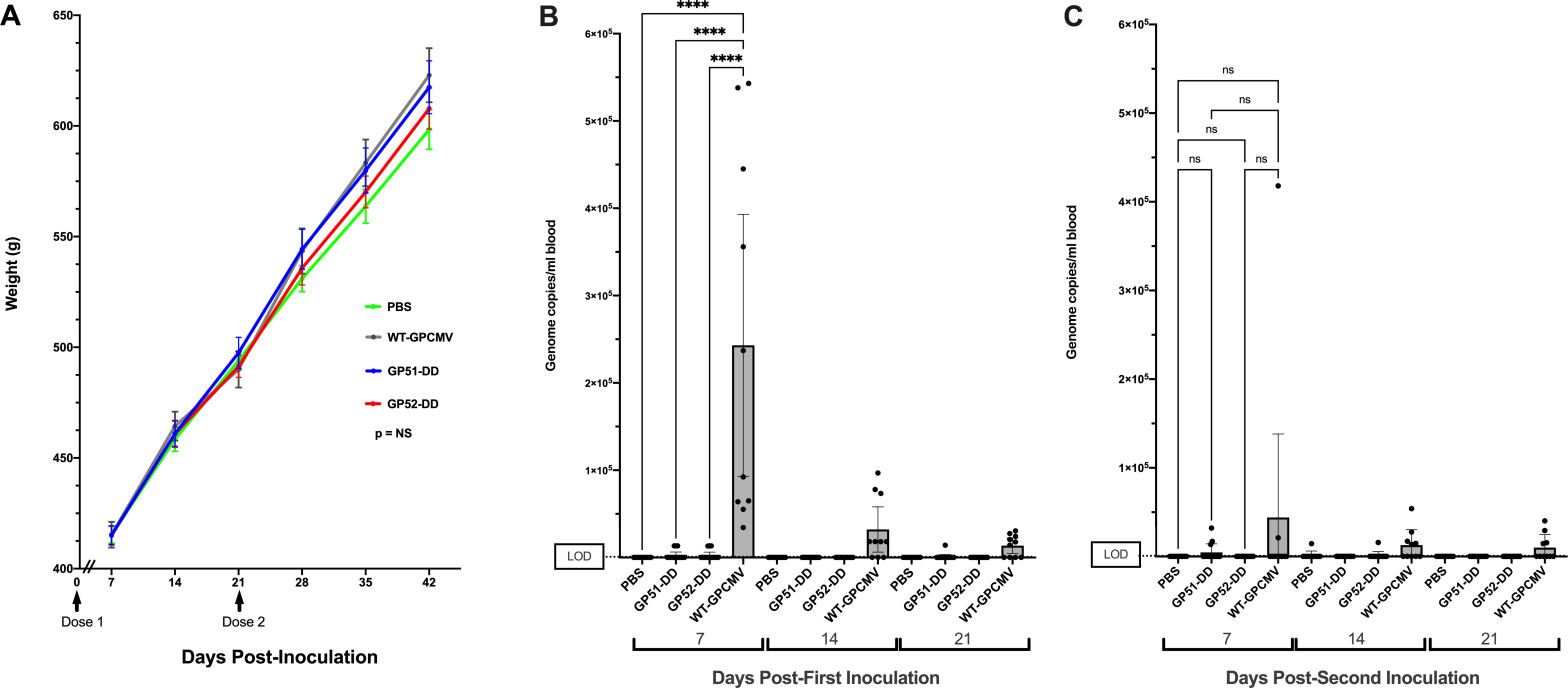
GP51-DD and GP52-DD are attenuated *in vivo* compared to wild-type guinea pig cytomegalovirus (GPCMV). Groups of female guinea pigs received two doses of GP51-DD or GP52-DD (*n* = 14), replication-competent WT-GPCMV (*n* = 10), or PBS (sham-inoculated) (*n* = 10). (**A**) Animal weights measured following inoculation of GP51-DD, GP52-DD, WT-GPCMV, or PBS. Animals were weighed at day 7 following initial inoculation of respective viruses and weekly thereafter. Timing of inoculation is shown by arrows. Weights represent mean ± SEM. Data shown were analyzed by repeated-measures ANOVA. None of the weight differences across the inoculation groups achieved statistical significance. (**B, C**) Assessment for DNAemia using blood samples collected 7, 14, or 21 days after the first (B) or second (C) inoculation that were assayed for GPCMV genome copy number by qPCR. Individual data points are shown with bars indicating mean\’[]s with their 95% confidence intervals (CI). Negative samples were assigned a value of 200 genome copies/mL of blood for statistical analyses, representing LOD of the assay (dotted line). By ANOVA, only at day 7 following first inoculation of wild-type virus was there a statistically significant difference in blood viral load compared to the GP51, GP52, or PBS (sham) inoculation groups (****P < 0.0001).

Immunization with GP51-DD, GP52-DD, or WT-GPCMV had no significant effects on guinea pig weight relative to animals that received sham inoculations (Fig. 2A), suggesting that the infectious doses used did not cause systemic illness. To determine whether GP51-DD or GP52-DD replicate *in vivo*, qPCR specific to GPCMV *GP83* was used to quantify DNAemia at day 7, 14, or 21 after each inoculation. Seven days after the first inoculation, DNAemia ranging in level from 3.4 × 10^4^ to 5.4 × 10^5^ genome copies/mL was detected in 10 of 10 animals inoculated with WT-GPCMV (Fig. 2B). By days 14 and 21 after first inoculation, 7 of 10 and 6 of 10 animals, respectively, remained DNAemic in the WT-GPCMV-inoculated group (Fig. 2B). In contrast, 3 of 14 animals inoculated with GP51-DD or GP52-DD were DNAemic at day 7 post-inoculation in each of these respective groups, with significantly lower mean levels of DNAemia compared to the WT-GPCMV group (*P* < 0.0001, Fig. 2B). A fourth animal in the GP51-DD group had low-level DNAemia at day 21 following the first inoculation. Following the second inoculation with these viruses, 3 of 14 animals in the GP51-DD group, 1 of 14 in the GP52-DD group, and 6 of 10 animals in the WT-GPCMV group were DNAemic at the time of analysis for at least one of the time points (days 7, 14, and 21) measured, although none of the viral loads compared in the respective groups were statistically significantly different across groups by ANOVA (Fig. 2C). In total, 10 of 28 animals (36%; 6 of 14 [43%] of the GP51-DD-inoculated animals and 4 of 14 [29%] of the GP52-DD-inoculated animals) had demonstrable DNAemia (*P* = 0.0005 compared to WT-GPCMV, Fisher’s exact test), albeit at levels marginally above the limit of detection of the assay.

### GP51-DD and GP52-DD vaccines induce humoral responses comparable to those conferred by WT-GPCMV infection

**Fig 3:**
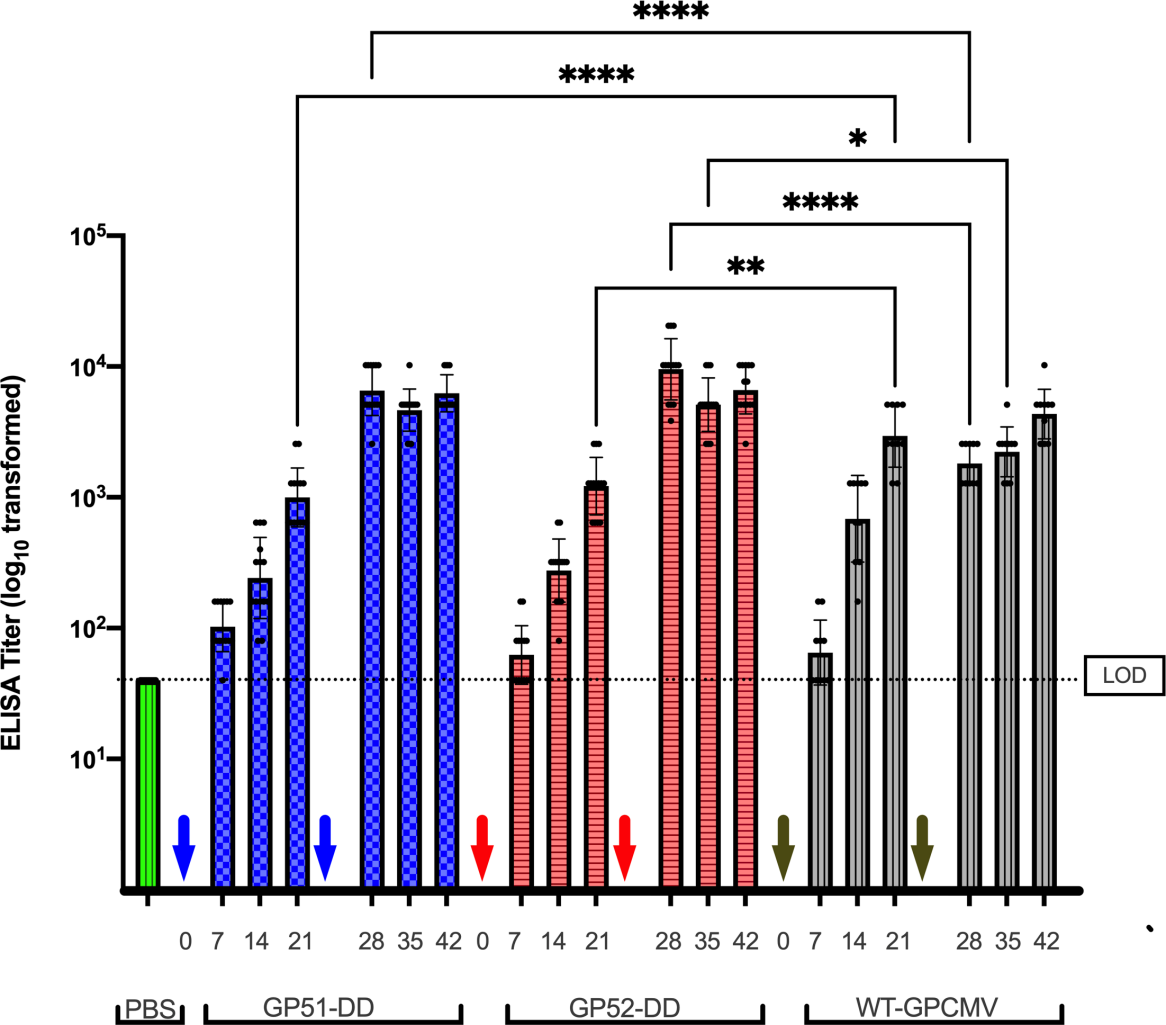
GP51-DD and GP52-DD vaccinations elicit similar IgG titers as WT-GPCMV vaccination. GPCMV-specific serum GMTs were measured by ELISA for GP51-DD (blue bars; *n* = 14), GP52-DD (red bars; *n* = 14), WT-GPCMV (gray bars; *n* = 10), or PBS (sham-inoculated, green bar [*n* = 10] animals, day 42 time point only). All PBS-immunized animals remained seronegative. The limit of detection for the ELISA is indicated by the dashed line. Arrows indicate days of first and second inoculations; the day 21 bleed was just prior to the administration of the second inoculation. Data shown represent analysis by repeated-measures ANOVA.

Antibody responses were next compared among the animal groups that had been immunized with GP51-DD, GP52-DD, and WT-GPCMV, or sham-inoculated with PBS. When GPCMV-reactive IgG titers were measured by ELISA (Table 2), the GMTs were similar for animals immunized with GP51-DD, GP52-DD, or WT-GPCMV at 7 and 14 days after the first dose. At day 21, the IgG titers of WT-GPCMV-immunized animals were higher than those of animals immunized with either GP51-DD (*P* < 0.0001) or GP52-DD (*P* < 0.01, Fig. 3). This trend reversed following the second vaccine dose, where it was noted that the IgG titers at day 28 in the WT-GPCMV-immunized animals were significantly lower (*P* < 0.0001) than those for animals immunized with GP51-DD or GP52-DD. The ELISA titer at day 35 in the vaccination sequence was also significantly higher in the GP52-DD vaccine group when compared to the WT-GPCMV group (*P* < 0.05).

Most of the inoculated guinea pigs were bred (see Materials and Methods), and pregnancy was established in 10 of 10 dams immunized with GP51-DD, nine of 10 dams immunized with GP52-DD, eight of eight dams immunized with WT-GPCMV, and 10 of 10 sham-inoculated controls (Table 2). Pregnant dams were challenged with WT-GPCMV at mid-gestation (approximately 35 days gestation), and blood was collected immediately before the gestational challenge (pre-challenge, Table 2) to evaluate for any decay in ELISA titer. Since dams became pregnant asynchronously, the viral challenge occurred variably between 65 and 98 days after the second vaccination.

**TABLE 2:** Group sizes^*a*^ and GPCMV-reactive IgG ELISA titers^*b*^

		GP51-DD		GP52-DD		WT-GPCMV		PBS	
	Day	*N*	Titer	*N*	Titer	*N*	Titer	*N*	Titer
First dose	7	14	102 ± 45	14	59 ± 43	10	65 ± 48	10	40 ± 0
	14		242 ± 206		276 ± 161		686 ± 477		40 ± 0
	21		999 ± 673		1,280 ± 771		3,152 ± 1,685		40 ± 0
Second dose	28	14	6,558 ± 2,872	14	9,547 ± 5,741	10	1,810 ± 675	10	40 ± 0
	35		4,637 ± 1,869		5,380 ± 2,546		2,229 ± 1,121		40 ± 0
	42		6,241 ± 2,400		6,751 ± 2,815		4,331 ± 2,262		40 ± 0
Pre-challenge	0	10	2,941 ± 1,383	9	2,765 ± 1,582	8	4,305 ± 2,600	10	43 ± 13

^*a*^ N, total animals per group.

^*b*^ GMT ± SD.

**Fig 4:**
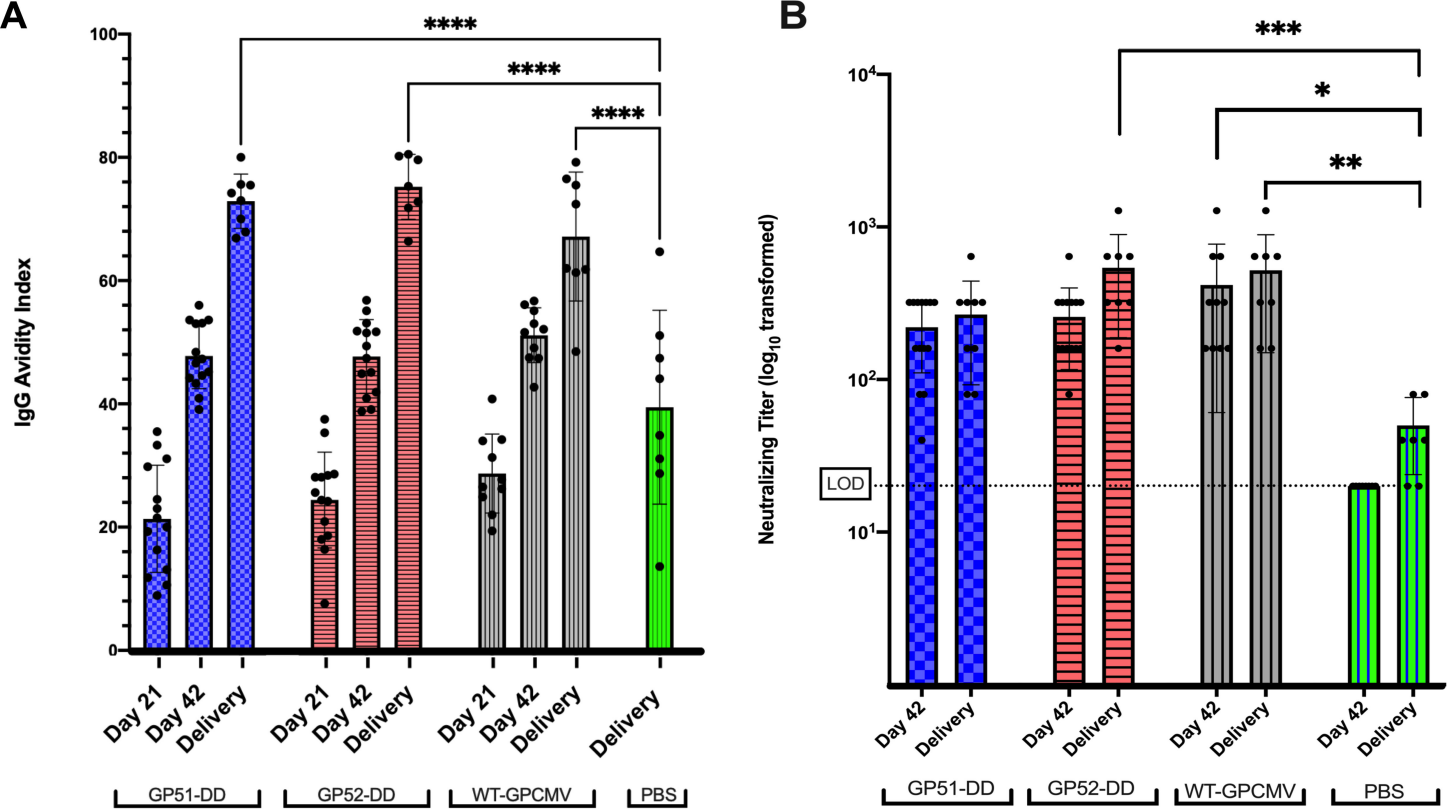
GP51-DD and GP52-DD vaccinations elicit similar IgG avidity and GPCMV neutralizing titers as WT-GPCMV vaccination. Guinea pigs were immunized as described previously and serum was collected 21 days after the first and second (day 42) doses and after delivery following viral challenge during pregnancy. (**A**) Avidity indices for sera from animals inoculated with GP51-DD, GP52-DD, WT-GPCMV, or PBS (sham-inoculated) were determined (****P < 0.0001). (**B**) Neutralizing titers were determined after the second doses or at delivery following challenge. Individual data points are shown, with bars indicating GMTs ± 1 SD and levels of significance (*P < 0.05; **P < 0.01; ***P < 0.001). PBS samples refer to the control (sham-immunized) animals; only sera obtained post-pregnancy challenge (at the time of delivery) demonstrated a response (limit of detection of neutralization assay is indicated by dashed line). Data shown represent analysis by repeated-measures ANOVA.

The IgG AI of GPCMV-reactive IgG in animals immunized with GP52-DD, GP51-DD, or WT-GPCMV was evaluated at the day 21 and 42 time points, and at delivery. AI increased progressively over the course of the experiment, and no statistically significant differences were observed between vaccine groups (Fig. 4A). Sham (PBS)-immunized dams seroconverted after the GPCMV challenge during pregnancy and had measurable AIs by the time of delivery; mean AIs of the GP52-DD, GP51-DD, and WT-GPCMV groups were statistically significantly higher (*P* < 0.0001) than that of the PBS group. Similarly, neutralizing antibody titers against GPCMV were not significantly different among groups immunized with two doses of GP51-DD, GP52-DD, or WT-GPCMV (Fig. 4B). Sham-immunized animals also developed neutralizing antibodies following the challenge with WT-GPCMV during pregnancy; however, at delivery, neutralizing potencies were significantly lower than those observed for challenged dams that had undergone preconception vaccination with GP52-DD or WT-GPCMV (*P* < 0.01). At day 42 post-preconception immunization, neutralizing titers in the WT-GPCMV inoculation group were also higher than those observed at delivery (post-challenge) in sham-immunized dams (*P* < 0.05; Fig. 4B). Taken together, these findings suggest that vaccination with either GP51-DD or GP52-DD elicited humoral responses that were comparable to WT-GPCMV infection in terms of the magnitude of the reactive-IgG response, the IgG AI, and the neutralizing potency, despite the apparent restricted capacities of GP51-DD and GP52-DD to replicate *in vivo*.

### Immunization with GP51-DD and GP52-DD elicits cell-mediated immunity (CMI) in animals inoculated with DD vaccines

**Fig 5:**
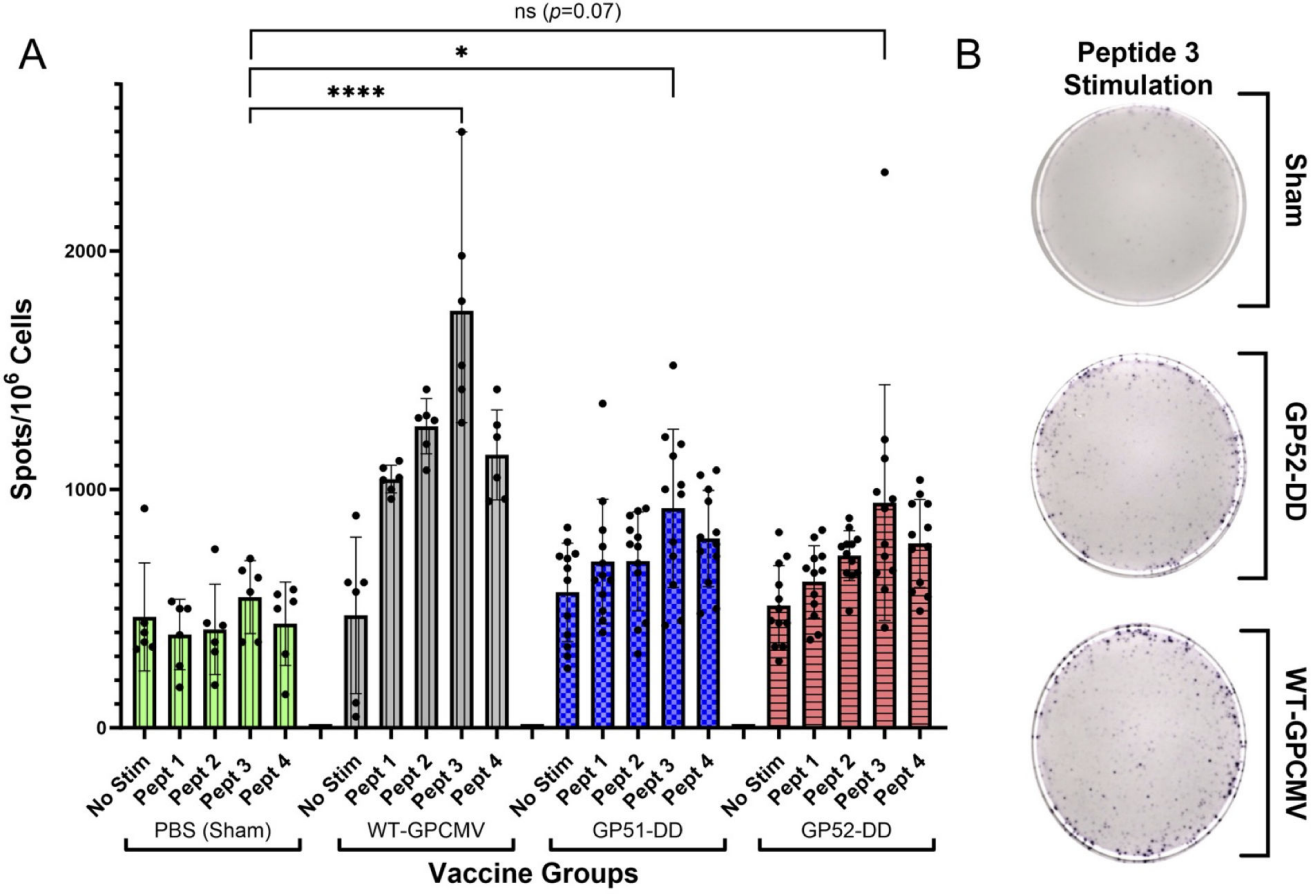
Vaccination with GP51-DD or GP52-DD induces CMI. An IFN-γ ELISpot assay was used to quantify CMI directed against four different GP83 peptides. (**A**) Results showing multiple replicates are shown by group for PBS (sham)-, WT-GPCMV-, GP51-DD-, and GP52-DD-immunized animals 28 to 35 days after completion of a two-dose vaccine series. The highest number of spots was observed using a peptide (LGIVHFFDN) corresponding to codons 333–341 of the GP83 ORF protein. Compared to PBS-immunized animals, the total spots were significantly higher in WT-GPCMV- (*P* < 0.0001) and GP51-DD-immunized (*P* < 0.05) animals. Significant differences in ELISpot responses to peptide 4 comparing sham-immunized animals with WT-GPCMV- (*P* < 0.001), GP-51-, and GP-52 immunized animals (both *P* < 0.01) were also observed. Statistically significant peptide 1- and 2-specific ELISpot responses were also observed in WT-GPCMV-immunized animals (*P* < 0.001 and *P* < 0.0001, respectively) compared to sham-vaccinated controls. (**B**) Representative ELISpot results are demonstrated as described in panel A.

To test the ability of the DD vaccines to elicit T-cell responses, a guinea pig-specific IFN-γ ELISpot was performed on a subset of immunized animals. Splenocytes were collected from guinea pigs following vaccination with PBS (sham-vaccinated control), WT-GPCMV, GP51-DD, and GP52-DD between 28 and 35 days after the second vaccination and before pregnancy was established. CMI responses against the T-cell immunogen and HCMV pp65 homolog, GP83, were measured (40). Previously, pooled peptide mapping studies of the T-cell response to GPCMV infection, as well as the response to immunization with GP83-specific vaccines, identified four peptides as key targets of the T-cell response. Using these peptides, we compared ELISpot responses in sham (PBS)-immunized animals and guinea pigs vaccinated with WT-GPCMV, GP51-DD, and GP52-DD. Significantly higher T cell responses to one or more of the peptides tested were noted in vaccinated animals compared to sham-immunized controls (Fig. 5). The highest number of spots was observed using peptide 3 (LGIVHFFDN), corresponding to codons 333–341 of GP83 (41). The total number of spots increased from 548 ± 153 (SD) in splenocytes from PBS-immunized animals to 1,748 ± 477 (SD) in WT-GPCMV-immunized animals (*P* < 0.0001), 921 ± 332 (SD) in GP51-DD immunized animals (*P* < 0.05), and 945 ± 494 (SD) in GP52-DD immunized animals (*P* = 0.07). Significant differences in ELISpot responses to peptide 4 comparing sham-immunized animals with WT-GPCMV- (*P* < 0.001), GP-51-, and GP-52-immunized animals (both *P* < 0.01) were also observed. Statistically significant peptide 1- and 2-specific ELISpot responses were also observed in WT-GPCMV-immunized animals (*P* < 0.001 and *P* < 0.0001, respectively) compared to sham-vaccinated controls.

### Immunization with GP51-DD and GP52-DD protects against maternal viremia and congenital GPCMV infection

**Fig 6:**
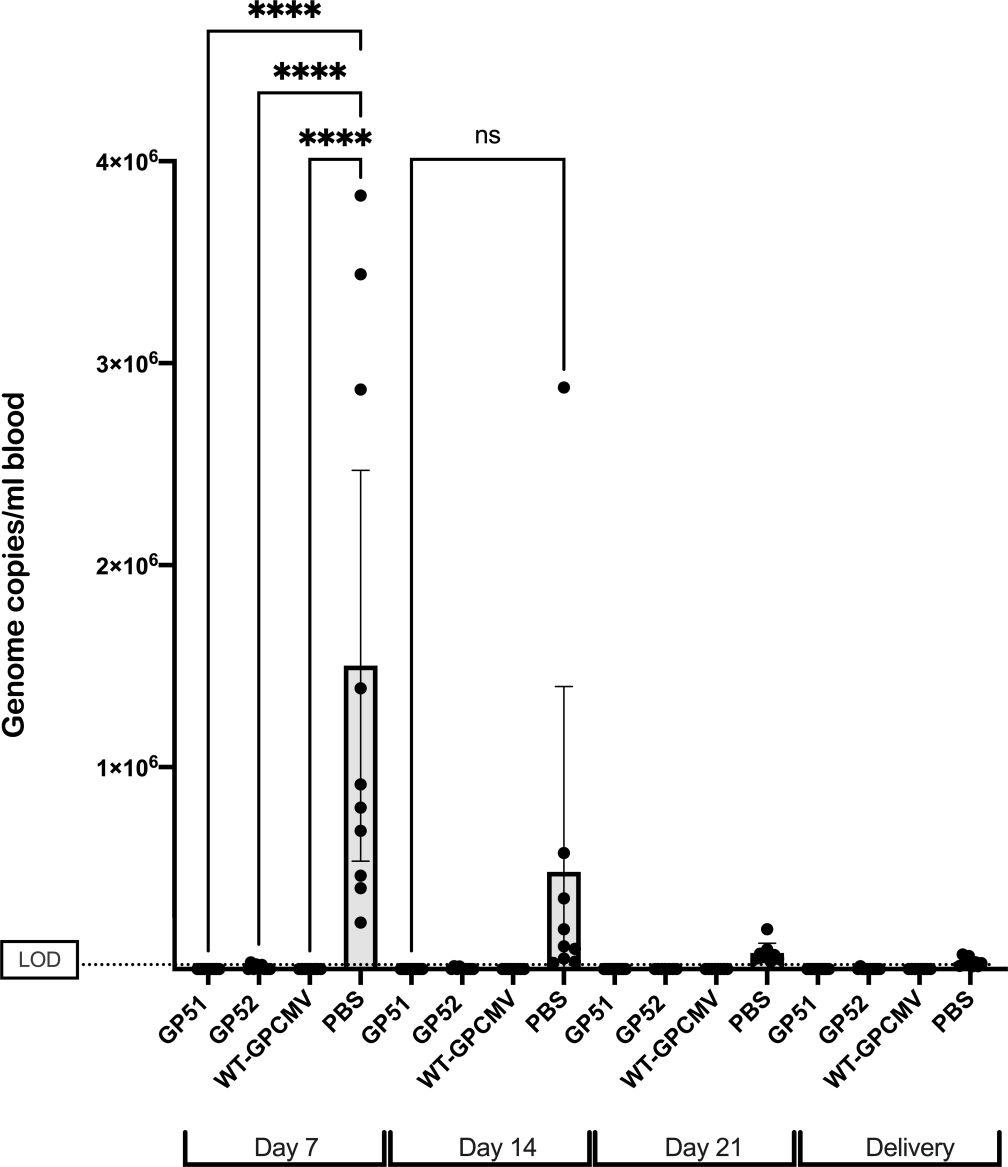
Vaccination reduces DNAemia in dams after GPCMV challenge during pregnancy. DNA was extracted from blood samples obtained from dams on days 7, 14, and 21 post-challenge and at delivery. DNAemia was assayed by qPCR. Individual data points are shown, with bars indicating means of log_10_-transformed data ± 95% CI (****P < 0.0001). Dashed line indicates the limit of detection of the assay.

We next compared the protective efficacy of GP51-DD and GP52-DD relative to either sham-inoculation or immunization with WT-GPCMV. All sham-inoculated (PBS-vaccinated) dams were DNAemic at most time points post-challenge, while GPCMV DNA was not detected at any time point in any of the dams that were immunized with GP51-DD or WT-GPCMV. Of the nine GP52-DD-immunized dams, three were DNAemic on day 7 post-challenge, and low but detectable levels of DNAemia were detected in two of these animals at day 14. DNAemia was also detected in one other GP52-DD-immunized dam at delivery (Fig. 6; Table 3). Thus, four of nine (44%) GP52-DD-immunized dams had DNAemia at one or more time points between the WT-GPCMV pregnancy challenge and delivery. The overall impact of preconception vaccination was that of a highly significant decrease in the magnitude of maternal DNAemia following viral challenge during pregnancy. Through ANOVA comparing all groups, the magnitude of DNAemia on day 7 was significantly lower in the GP51-DD-, GP52-DD-, and WT-GPCMV-immunized dams (*P* < 0.0001) compared to the sham-inoculated animals (Fig. 6).

**TABLE 3:** Incidence of maternal DNAemia^*a*^ following challenge of pregnant, vaccinated dams

Day	GP51-DD		GP52-DD		WT-GPCMV		PBS	
	Incidence	%	Incidence	%	Incidence	%	Incidence	%
7	0/10	0	3/9	33	0/8	0	10/10	100
14	0/10	0	2/9	22	0/8	0	9/9^*c*^	100
21	0/10	0	0/7^*d*^	0	0/8	0	9/9	100
Delivery	0/10	0	1/6^*e*^	17	0/8	0	8/8^*f*^	100
Combined*^b^*	0/10	0	4/9	44	0/8	0	10/10	100

^*a*^ Dams with a PCR^+^ result in blood/total evaluable at that time point.

^*b*^ Dams with at least one PCR^+^ result in blood at any time point/total.

^*c*^ One dam in this group delivered before day 14.

^*d*^ Two dams in this group delivered before day 21.

^*e*^ Viremia samples were obtained from only six dams in this group upon delivery.

^*f*^ Viremia samples were obtained from only eight dams in this group upon delivery.

**Fig 7:**
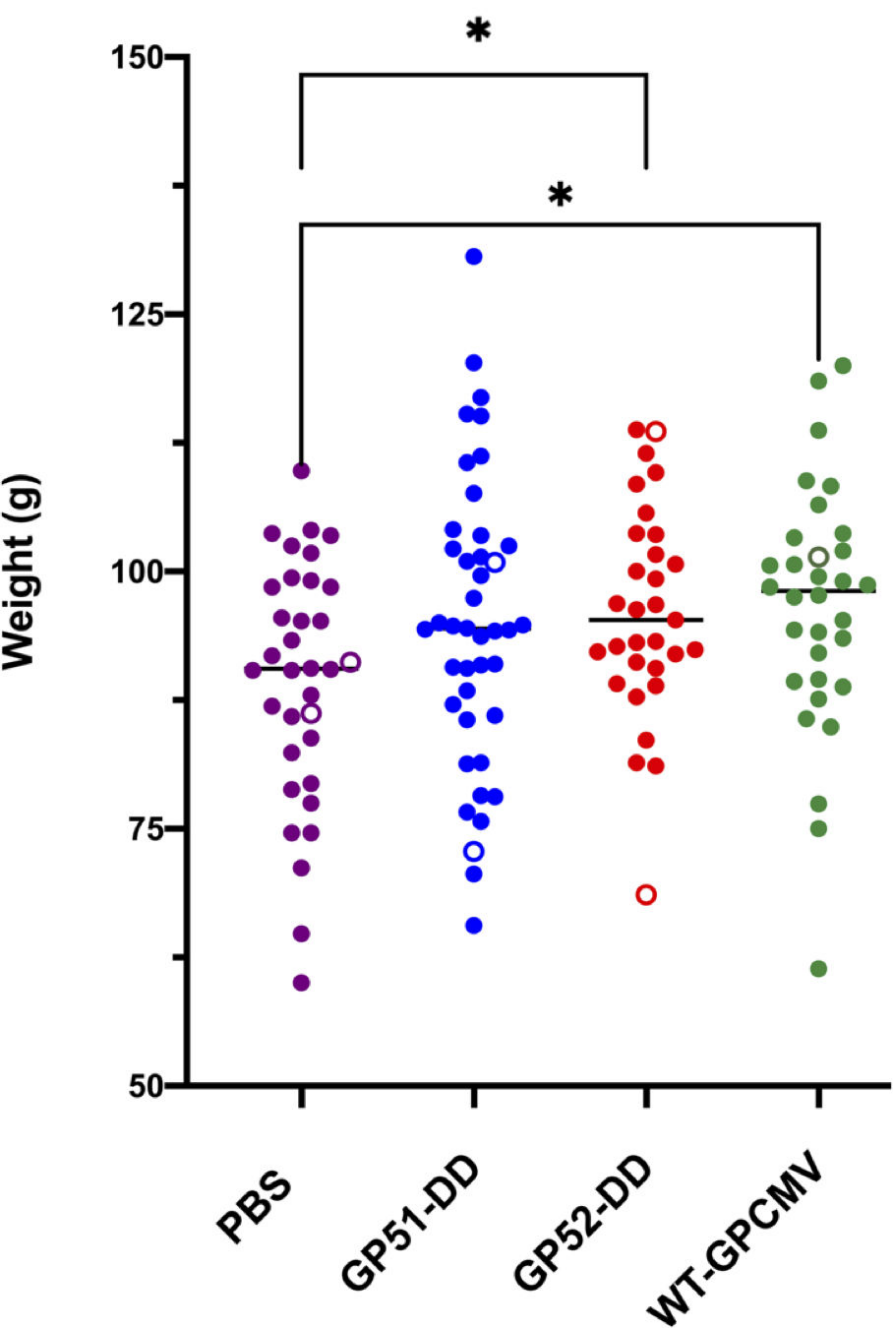
Pre-conception maternal vaccination results in improved pup birth weights after the GPCMV challenge during pregnancy. Individual pup weights at birth are shown with maternal vaccine groups color-coded and stillborn pups indicated by open circles. For statistical comparison, pups from dams that received GP51-DD or GP52-DD vaccines or WT-GPCMV were analyzed in paired comparisons to the PBS (sham-inoculated) group by *t*-test, with significant differences indicated (*P < 0.05).

Next, the duration of the pregnancies post-virulent WT-GPCMV challenge of dams, and the overall rates of pup mortality in the various groups, were compared (Table 4). No statistically significant differences were noted in pregnancy durations or pup mortality. However, the mean birth weights were lower for pups born to sham-inoculated dams compared to both pups born to the WT-GPCMV group and pups born to the GP52-DD-immunized group (Fig. 7; *P* < 0.05).

**TABLE 4:** Pregnancy duration post-challenge, pup infection, and pup mortality across groups

Finding		GP51-DD		GP52-DD		WT-GPCMV		PBS	
Pregnancy duration^*a*^		28.8 ± 3.5		26.8 ± 6.8		29.4 ± 5.1		29.4 ± 9.2	
		Incidence	%	Incidence	%	Incidence	%	Incidence	%
Infection^*b*^	Blood	1/42	2.4	1/31	3.2	0/32	0	9/34	26
	Lung	2/42	4.8	2/31	6.5	0/32	0	25/34	74
	Liver	1/42	2.4	2/31	6.5	0/31	0	20/34	59
	Spleen	1/42	2.4	2/31	6.5	0/31	0	30/34	88
	Combined^*c*^	2/39	5.1	3/31	9.7	0/32	0	31/34	91
Mortality^*d*^		2/37	5.4	2/29	6.9	1/31	3.2	2/32	6.3

^*a*^ Days; *P* = NS across groups.

^*b*^ Pups with a PCR^+^ result in samples indicated (blood or organs)/total.

^*c*^ Pups with at least one PCR+ result in any sample (blood or organs)/total.

^*d*^ Stillborn pups/total.

**Fig 8:**
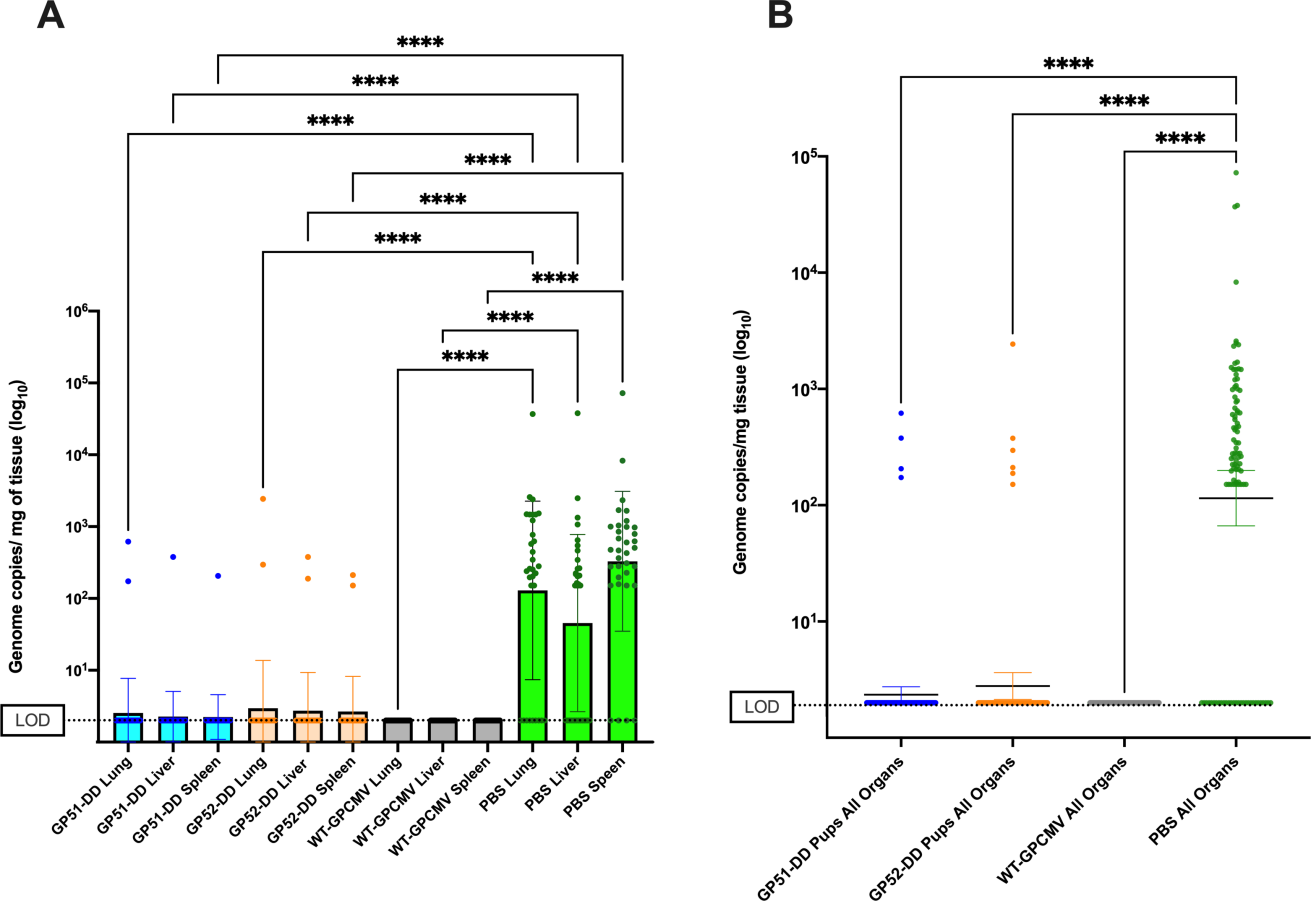
Immunization with GP51-DD or GP52-DD significantly reduces viral loads in pup organs. DNA was extracted from pup blood and tissues after delivery, and viral loads were quantified by qPCR. (**A**) PCR results for pup visceral tissue (lung, liver, spleen) for each vaccine group are compared by organ. Samples below the LOD for the PCR assay (dashed line) were assigned a value of two genome copies/mg for statistical comparisons. (**B**) Results for all visceral organ samples (lung, liver, and spleen) combined for comparisons of each individual vaccine group. Data shown are means of log_10_-transformed viral load data ± 1 SD compared by one-way ANOVA. ****P < 0.0001.

Finally, DNA was extracted from pup blood and tissues to assay for congenital infection (Table 4). Congenital infection was defined as any positive PCR from pup blood or visceral organ (lung, liver, or spleen) using samples obtained from pup necropsy within 24 h of delivery. Using this definition, we noted that 31 of 34 pups (91%) born to sham-inoculated dams had congenital GPCMV infection, as evidenced by PCR detection of viral DNA in blood, lung, liver, or spleen. In contrast, viral DNA was observed in two of 39, three of 31, and none of 32 pups born to dams immunized with GP51-DD, GP52-DD, or WT-GPCMV, resulting in protective efficacies of 94, 89, and 100%, respectively (Fig. 8A; Table 4). Notably, one of the four DNAemic, GP52-DD-immunized dams gave birth to three pups, two of which had congenital GPCMV infection. Congenital infection did not occur in the three remaining DNAemic dams. A third pup in the GP52-DD group had congenital GPCMV infection (detected in blood only), although the corresponding maternal PCRs were all negative. The differences for each vaccine group by organ are shown in Fig. 8A. When viral loads were compared individually for the lung, liver, and spleen, highly significant viral load reductions (*P* < 0.00001) were noted for each vaccine approach compared to the sham (PBS)-immunized group. When viral loads were combined for all tissues in aggregate and compared (Fig. 8B), a significant overall reduction (*P* < 0.0001) in the pup viral load was also noted.

## DISCUSSION

A vaccine for the prevention of congenital HCMV infection is a major public health priority (42), but the ideal platform for such a vaccine is uncertain. Subunit vaccines based on the immunodominant gB have been evaluated in multiple clinical trials; these trials have demonstrated no better than ~45–50% efficacy in preventing primary infection in women of childbearing age (43, 44). Although the correlates of immunity required for protection of the fetus following maternal infection and/or immunization are unknown (45), it may be that a broader repertoire of immune responses—beyond anti-gB responses—is required. Such a goal could be achieved with a whole virus HCMV vaccine. The first HCMV vaccine candidates were comprised of live strain Towne or AD169, and both were produced following extensive serial passage on fibroblasts (46–49). Although these vaccines were highly attenuated when administered in human clinical trials, a phase 2 trial of the Towne vaccine in young women with children attending group day-care centers—a high-risk population for acquisition of a primary HCMV infection—did not demonstrate vaccine efficacy (50). Moreover, a low-passage strain, Toledo, was pathogenic in naturally infected or Towne-vaccinated humans, even following administration of as few as 100 PFU (51). Thus, lack of evidence for efficacy and concerns about safety have dampened enthusiasm for further development of live-attenuated HCMV vaccines.

Replication-defective virus vaccines (including disabled, infectious, single-cycle or “DISC” vaccines) represent an immunization strategy that, in principle, retains the benefits of a live virus vaccine while ensuring safety. For example, a whole virus GPCMV DISC vaccine rendered replication-defective by a mutation disrupting *GP85*, an essential capsid-encoding gene and the homolog of *UL85*, elicited a broad repertoire of humoral and cellular responses and provided a high level of protection against congenital transmission (52, 53). However, in the rhesus macaque model, a whole virus vaccine rendered replication-defective by the deletion of sequences encoding glycoprotein L and several additional genes involved in immune evasion was less effective than a soluble recombinant gB protein vaccine at reducing post-challenge DNAemia (54). While the replication-defective vaccine was superior in inducing CMI, it elicited less robust humoral immunity both with respect to viral antigen-reactive and neutralizing antibodies (54). Thus, at least in the rhesus model, the replication-defective strategy did not confer any clear advantage over the gB subunit vaccine.

In this study, we examined two recombinant GPCMV vaccines where essential viral proteins were modified using the same protein destabilization technology used in V160. For both the GP51-DD and GP52-DD vaccines, a few animals exhibited low-level DNAemia following immunization. This was not surprising in the case of GP51-DD, which replicates *in vitro* without Shield-1 (Fig. 1B). GP52-DD was much more dependent on the presence of Shield-1 for replication, though very low levels of replication may occur *in vivo* and *in vitro*. In the experiment shown in Fig. 1C, small foci of infected cells were observed in the cell monolayer, and in a replicate experiment at a higher MOI, very low levels of cell-free infectious virus (~20 PFU/mL) were detected at 14 days post-infection. The low-level DNAemia that was detected could also be an artifact of DNA from the vaccine inoculum or DNA produced by cells unproductively infected by virions in the vaccine that gradually reached the blood. The difference in the Shield-1 dependence of GP52-DD vs. GP51-DD was unexpected, and future experiments are required to reconcile this. The effectiveness with which different proteins are destabilized by fusion to DD varies widely, as does the ability of Shield-1 to stabilize different DD fusions (22, 39). Presumably, differences in the stability of GP52-DD and GP51-DD are reflected in the different Shield-1 dependence of these viruses. However, we have no experimental data to support this, in part because we lack antibodies to test the stability of GP51-DD/GP52-DD proteins. Even so, we consider both GP51-DD and GP52-DD vaccines *replication-deficient* rather than fully replication-defective. As in V160, it may be necessary to target multiple essential viral proteins for degradation to generate a fully replication-defective GPCMV DISC vaccine.

Despite evidence that GP52-DD is far more replication-deficient than GP51-DD *in vitro*, GP52-DD was similarly immunogenic to GP51-DD. Both replication-deficient vaccines compared favorably with fully replication-competent WT-GPCMV with respect to the induction of total GPCMV-reactive IgG, IgG avidity maturation, and virus-neutralizing activities. Thus, the ability to replicate efficiently *in vivo* is not a requirement for vaccine immunogenicity, at least insofar as the immune parameters that were measured.

Preconception vaccination with WT-GPCMV appeared to induce sterilizing immunity for dams that underwent subsequent viral challenge during pregnancy. While post-challenge DNAemia was also not detected in dams vaccinated with GP51-DD, the presence of viral DNA in two pups born to GP51-DD-immunized dams suggests that GP51-DD vaccination did not provide complete sterilizing maternal immunity. GP52-DD was partially protective, as 44% of immunized dams exhibited low-level post-challenge DNAemia, while the remaining 56% were negative at all time points. Vaccination with GP52-DD and WT-GPCMV slightly improved pup birthweights compared to sham vaccination, but no differences were observed with respect to the duration of pregnancy and magnitude of pup mortality, which for all groups were on par with that of unchallenged pregnancies (55).

When pups were examined for evidence of intrauterine infection by PCR, 91% of pups born to sham-inoculated dams were positive in at least one sample, indicating efficient vertical transmission in the absence of preexisting maternal immunity. Vaccination with WT-GPCMV fully prevented vertical transmission, while vaccination with GP52-DD or GP51-DD reduced but did not eliminate transmission, resulting in 9.7% or 5.1% of pups infected in these groups, respectively, corresponding to a protective efficacy of 89% or 94%.

Our study findings in guinea pigs are applicable to the development of a vaccine to prevent congenital HCMV transmission. A replication-defective HCMV vaccine, V160, was recently evaluated in human clinical trials (21, 56–58). The vaccine is based on the AD169 strain modified to express UL51, IE1, and IE2 as N-terminal DD fusions. In addition, a mutation in *UL131A* was repaired to restore expression of the HCMV PC (21, 57, 58). Safety and immunogenicity of V160 were confirmed in a phase 1 study, and neutralizing antibody titers induced by V160 vaccination in CMV-seronegative individuals were similar to those observed with natural infection (59). This included antibody responses that specifically neutralized epithelial cell entry (59) that were deficient in subjects vaccinated with gB/MF59 subunit vaccine or the live Towne vaccine, presumably because neither are able to induce antibodies specific to the PC (60).

In a recent phase 2b study, a three-dose series of the V160 vaccine demonstrated an efficacy of 44.6% against acquisition of primary HCMV infection in young seronegative women (19). However, because the serological detection of naturally acquired infections in the context of antibodies induced by V160 vaccination was not feasible, PCR was used, and, surprisingly, a significant number of placebo recipients were PCR-positive but never developed CMV-reactive antibodies. Thus, some PCR-positive V160 recipients may similarly have been false positives (61). Based on this assumption and correcting for false positives among the V160 recipients, efficacy increased to 67 or 77% depending on the vaccine group (19).

While there are limits to which the findings reported in the current study can inform the development of HCMV vaccines, two aspects are worth noting. First, immunity induced by WT-GPCMV was more protective against maternal DNAemia than that induced by GP52-DD and more protective against vertical transmission than either GP51-DD or GP52-DD. However, all three immunizations induced similar humoral responses. This suggests that other aspects of the adaptive response to WT-GPCMV are necessary to prevent 100% of fetal infections. Such components include T-cells (62), as well as non-neutralizing, Fc-mediated humoral responses, such as those proposed for HCMV (63–66). Moreover, as the breadth of humoral responses to the spectrum of GPCMV antigens was not examined, GP51-DD or GP52-DD may have inadequately induced antibodies to specific viral proteins that are important for sterilizing immunity. Thus, further efforts to identify immune parameters that are elevated following WT-GPCMV compared to GP51-DD or GP52-DD vaccination are warranted.

Second*,* while the 89% efficacy against fetal infection by the more replication-deficient GP52-DD vaccine is encouraging, efficacy against maternal infection was modest and on par with that reported for V160 in humans (19). As maternal infection will likely be the primary endpoint for approval of a candidate HCMV vaccine (67), there is room for improvement of replication-defective vaccines over the benchmark set by V160. The design of V160 could potentially be improved in several ways. For example, given the plethora of genetic defects inherent in the AD169 background used for V160 (68, 69), one potential improvement could be to base a replication-defective vaccine on a wild-type HCMV, such as the Merlin strain (70). A second potential deficiency arises from destabilization of IE1 and IE2 through DD fusions (21), as *in vivo*, degradation of IE1 and IE2 likely precludes V160-infected cells from expressing late viral proteins, including many important targets of humoral and cellular immunity. Thus, a replication-defective vaccine, in which only late proteins are destabilized, might be far more immunogenic. Lastly, numerous strategies could be employed to further enhance the immunogenicity of a replication-defective vaccine, for example, through insertion of gene cassettes to hyper-express certain viral immunogens or by deletion of viral genes that encode proteins having immune-modulatory functions.

The current GPCMV study had other limitations that may temper the applicability of the findings to HCMV vaccines. In this study, immunization/infection with WT-GPCMV virus induced essentially sterilizing immunity, preventing maternal re-infection and congenital GPCMV transmission upon subsequent challenge. Although vaccination with wild-type virus conferred complete sterilizing immunity to congenital GPCMV transmission, it is clear that natural immunity only partially protects against superinfection/reinfection in humans (71, 72) and rhesus macaques (73). The reasons for this require further investigation but may be related to the animal model, the relatively short time between immunization and challenge, or other factors.

Another potential limitation of this study is that the vaccine response read-outs focused primarily on the measurement of antibody responses, including neutralizing antibody titers and IgG AI. However, a subset of vaccinated animals was evaluated for CMI responses by IFN-γ ELISpot. It was of interest that CMI responses were generated, even in the absence of replication competence (in the case of GP52-DD), but these studies were performed in a small number of non-pregnant animals and not in vaccinated dams that completed pregnancy following mid-gestation GPCMV challenge. Future studies should focus in greater detail on enumerating the potential role of vaccine-engendered T-cell responses, in particular CD4+ T cells, in the context of pregnancy to gauge the role of cellular immunity in preventing vertical viral transmission (74). As noted above, many other functions are important for protection against HCMV infection, including congenital CMV infection. These include non-neutralizing, Fc-mediated mechanisms, such as antibody-dependent cellular phagocytosis or antibody-dependent cellular cytotoxicity (63, 65, 66). Another limitation of this study was that the challenge for dams was performed via a parenteral route of inoculation, whereas HCMV infections are predominantly acquired at mucosal surfaces (75). Future GPCMV studies addressing these points will increase the relevance of this small animal model to the testing of HCMV vaccines against congenital infection.

Although an HCMV mRNA vaccine is in the advanced stages of clinical trials (3), no vaccine is yet licensed for prevention of HCMV infection in women of childbearing age. Despite sub-optimal efficacy reports from an initial clinical trial (19), efforts to improve immunogenicity and enhance the efficacy of replication-defective vaccines are warranted. The results presented here demonstrate that a whole virus GPCMV vaccine that is highly replication-deficient provides significant levels of protection against maternal and congenital infection. Thus, a fully replication-defective GPCMV that emulates V160 while avoiding the IE destabilization that is a feature of that vaccine could be used as a platform to explore modifications designed to enhance immunogenicity and protective efficacy against maternal infection and vertical transmission. Additionally, while sterilizing immunity may be necessary to prevent maternal infection, a lower standard may be sufficient to prevent congenital transmission and, more importantly, HCMV disease in the infant. Future experiments aimed at modeling prevention of the disease sequelae of congenital GPCMV infection, including studies of SNHL (76) and neurodevelopment (24), can provide insights into how even a modestly effective HCMV vaccine could improve newborn outcomes, even if sterilizing immunity is not achieved.

## Data Availability

The RFP-tagged GPCMV, N13R10r129-RFP, was sequenced during the course of these studies and assigned GenBank accession number PQ384583.1. These data are available at the following URL: https://www.ncbi.nlm.nih.gov/nuccore/PQ384583.1.
